# The Pub1 and Upf1 Proteins Act in Concert to Protect Yeast from Toxicity of the [*PSI*^+^] Prion

**DOI:** 10.3390/ijms19113663

**Published:** 2018-11-20

**Authors:** Valery N. Urakov, Olga V. Mitkevich, Alexander A. Dergalev, Michael D. Ter-Avanesyan

**Affiliations:** Bach Institute of Biochemistry, Federal Research Center “Fundamentals of Biotechnology” of the Russian Academy of Sciences, 119071 Moscow, Russia; valery.urakov@gmail.com (V.N.U.); alexanderdergalioff@gmail.com (A.A.D.)

**Keywords:** *Saccharomyces cerevisiae*, [*PSI*^+^] prion toxicity, translation termination factors, Sup35, Sup45, Pub1, Upf1

## Abstract

The [*PSI*^+^] nonsense-suppressor determinant of *Saccharomyces cerevisiae* is based on the formation of heritable amyloids of the Sup35 (eRF3) translation termination factor. [*PSI*^+^] amyloids have variants differing in amyloid structure and in the strength of the suppressor phenotype. The appearance of [*PSI*^+^], its propagation and manifestation depend primarily on chaperones. Besides chaperones, the Upf1/2/3, Siw14 and Arg82 proteins restrict [*PSI*^+^] formation, while Sla2 can prevent [*PSI*^+^] toxicity. Here, we identify two more non-chaperone proteins involved in [*PSI*^+^] detoxification. We show that simultaneous lack of the Pub1 and Upf1 proteins is lethal to cells harboring [*PSI*^+^] variants with a strong, but not with a weak, suppressor phenotype. This lethality is caused by excessive depletion of the Sup45 (eRF1) termination factor due to its sequestration into Sup35 polymers. We also show that Pub1 acts to restrict excessive Sup35 prion polymerization, while Upf1 interferes with Sup45 binding to Sup35 polymers. These data allow consideration of the Pub1 and Upf1 proteins as a novel [*PSI*^+^] detoxification system.

## 1. Introduction

Similar to other amyloids, most prions are formed in a process of highly ordered non-covalent polymerization of partially misfolded protein monomers. The ability to form amyloids is a common inherent feature of conformationally flexible proteins, which in many cases contain intrinsically disordered domains and, since such proteins are widespread in nature, amyloids are found in a wide range of organisms from mammals to bacteria, where they can have both deleterious and beneficial effects [1,2]. While in mammals, prions cause neurodegenerative diseases, in fungi they mediate non-chromosomal inheritance of several phenotypic traits [3,4]. Importantly, due to the high genetic tractability of *Saccharomyces cerevisiae*, its prions, and especially [*PSI*^+^], are the most well studied. [*PSI*^+^] is a prion determinant that gives rise to a nonsense suppressor phenotype as a consequence of the amyloid aggregation and partial inactivation of the translation termination factor Sup35 (eRF3) [5,6,7]. Prionization of Sup35 can result in appearance of multiple [*PSI*^+^] variants that differ by the strength of their nonsense suppressor phenotype and stability of inheritance [8,9]. The dissimilarity in the properties of [*PSI*^+^] variants reflects heritable differences in the structure of Sup35 prion polymers [10,11]. Although the process of prion polymerization is autocatalytic, in vivo the appearance of [*PSI*^+^], as well as its propagation and manifestation depend on the activity of chaperones (for a review, see Reference [12]). Besides chaperones, some non-chaperone proteins interacting with prion-forming proteins can also influence the properties of prion amyloids. For example, Sla1-mediated interaction of Sup35 with the actin cytoskeleton was shown to promote generation of the [*PSI*^+^] prion [13]. The interaction of Sup35 with the Sup45 (eRF1) termination factor has two effects, it decreases prion formation [14] and it can contribute to [*PSI*^+^] toxicity [15,16].

The biological significance of yeast prions, and the [*PSI*^+^] prion in particular, is still subject to debate [17,18,19,20,21,22]. Here, we confirm the role of Pub1 in Sup35 polymerization [23] and also show that the Upf1 protein, involved in the control of the nonsense codon-mediated mRNA decay (NMD) [24], decreases sequestration of Sup45 into Sup35 prion polymers. Individually, the Pub1 and Upf1 proteins do not alleviate the harmful effects of [*PSI*^+^], but their combined action can suppress [*PSI*^+^] toxicity and, therefore, these proteins can be viewed as a [*PSI*^+^] detoxification system.

## 2. Results

### 2.1. Simultaneous Deletion of PUB1 and UPF1 in the Presence of [PSI^+^] Can Be Synthetic Lethal

The work was inspired by an incidental observation made during elucidation of the role of Pub1 in translation termination [23], demonstrating that the *UPF1* gene could be deleted in the 74-D694 strain with deleted *PUB1*, only if this strain did not carry strong [*PSI*^+^]_S7_. This indicated that simultaneous deletion of *PUB1* (*pub1-Δ*) and *UPF1* (*upf1-Δ*) in the presence of [*PSI*^+^] caused synthetic lethality. To confirm this, we deleted *UPF1* in the transformants of 74-D694 [*PSI*^+^]_S7_ deleted for *PUB1*, which carry the wild-type *PUB1* gene on multicopy plasmids with either *LEU2* or *URA3*. In the obtained strains, these plasmids could not be changed for the empty vectors with complementary markers, though they were easily interchangeable for the multicopy *PUB1* or *UPF1* plasmids with appropriate selectable markers. (Table 1). These experiments showed that the 74-D694 strain with *PUB1* and *UPF1* deletions could grow only if it did not contain [*PSI*^+^]_S7_ or expressed plasmid-encoded copies of the *PUB1* or *UPF1* genes.

**Figure 1:**
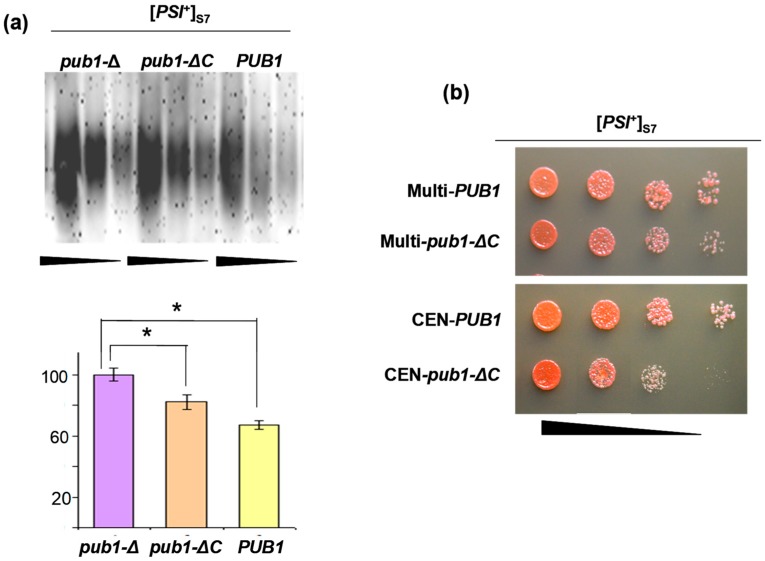
Plasmid-encoded Pub1∆C slightly compensates the effect of chromosomal *pub1-∆* on prion polymerization of Sup35 in [*PSI*^+^]_S7_ cells and alleviates the synthetic lethal interaction between *pub1-Δ*, *upf1-Δ* and [*PSI*^+^]_S7_. (**a**) SDD-AGE analysis of polymerized Sup35 in transformants of the 74-D694 [*PSI*^+^]_S7_ strain, with *PUB1* deletion carrying multicopy plasmids encoding wild-type Pub1 (*PUB1*), Pub1∆C (*pub1-∆C*), or empty vector (*pub1-∆*). The transformants were grown in liquid Sc-Ura medium selective for the plasmid marker. Blots were probed with the polyclonal antibody against Sup35NM. Equal amounts of total protein from the compared cell lysates were serially diluted in two-fold decrements. Undiluted samples contained ~180 µg of total protein per lane. Four independent transformants of each type were analyzed and representative blot images are presented. Abundances of polymerized Sup35 (percent of the level in the *pub1-∆* strain ± SEM) were calculated after densitometry of blots and are shown on the histograms. Statistically significant differences of polymerized Sup35 in compared transformants (marked by trapezoid), determined by Student’s *t*-test, are indicated by asterisks (* *p* < 0.05). (**b**) Growth of the transformants on the solid SC-Ura medium. The transformants of the *pub1-Δ upf1-Δ* [*PSI*^+^]_S7_ strain with multicopy YEplac195-PUB1 (Multi-*PUB1*), YEplac195-PUB1ΔC (Multi-*pub1-ΔC*) and centromeric pRS316-PUB1 (CEN-*PUB1*), pRS316-PUB1ΔC (CEN-*pub1-ΔC*) plasmids were grown in liquid SC-Ura medium and after 12 h incubation, cell suspensions were diluted to an OD_600_ of 0.3, spotted onto plates with the same medium and incubated for four days at 30 °C. Four serial three-fold dilutions of cell suspensions are shown.

In contrast to *UPF1*, the molecular mechanism responsible for the *PUB1* rescuing effect is clear, since adding a *PUB1* wild-type allele prevents the increase of Sup35 polymerization caused by *pub1-Δ*, which is most probably due to the ability of Pub1 to interact with Sup35 [23]. However, to our surprise, the plasmids encoding the Pub1 variant without a C-terminal extension (Pub1∆C) which contains the major site through which Pub1 interacts with Sup35, also suppressed synthetic lethality. This suggested that weak interaction with Sup35 mediated by the Pub1 internal low-affinity binding site [23] was sufficient for inhibition of Sup35 polymerization. To test this suggestion, we compared the amount of Sup35 polymers in the 74-D694 [*PSI*^+^]_S7_ strain containing chromosomal *pub1-∆* and plasmids with either *pub1-∆C* or wild-type *PUB1*, or an empty vector. In accordance with our suggestion, *pub1-∆C* caused a small, but statistically significant decrease in the amount of Sup35 polymers (Figure 1a). Importantly, suppression of synthetic lethality by *pub1-∆C* was also incomplete, as revealed by a decreased growth rate of transformants carrying the *pub1-∆C* plasmid compared to growth of transformants with the plasmid bearing wild-type *PUB1* (Figure 1b). It is also notable that although we showed earlier that *pub1-Δ* causes an approximately 2-fold increase in the amount of Sup35 polymers in the cells with [*PSI*^+^]_S7_ [23], in this work this difference was only about 1.5-fold. This discrepancy could be due to different growth conditions. Indeed, in an earlier work we compared strains with the chromosomal *pub1-Δ* and *PUB1* alleles, grown in rich YPD medium (see Materials and Methods), whereas here we examined levels of Sup35 polymers in transformants grown in synthetic medium selective for the plasmid marker.

Next, we examined the ability of the Sup35C protein lacking the N-terminal prion-forming domain to suppress synthetic lethal interaction between *pub1-∆*, *upf1-∆* and [*PSI*^+^]_S7_. It is known that due to the absence of the prion domain, Sup35C cannot polymerize in [*PSI*^+^] cells, though it retains the ability to bind Sup45, thus interfering with sequestration of Sup45 into Sup35 polymers [5,25]. However, only multicopy *sup35-C* plasmid ensured cell viability, thus suggesting that high levels of soluble Sup35C were required for sufficient binding of Sup45, which in turn prevents its sequestration. Finally, the role of Sup45 depletion in synthetic lethality was proved by the ability of its overproduction to rescue lethality of the *pub1-∆ upf1-∆* [*PSI*^+^]_S7_ strain (Table 1).

It should be noted that *UPF1* controls NMD in concert with the *UPF2* and *UPF3* genes and deletion of any one of them completely abolishes decay of nonsense-containing mRNAs [26]. Importantly, besides NMD, these genes also control nonsense codon readthrough, and deletion of each of them increases readthrough to approximately the same level [27]. However, despite this functional similarity, deletion of either *UPF2* or *UPF3*, as well as simultaneous deletion of these genes in the 74-D694 [*PSI*^+^]_S7_ strain deleted for *PUB1* did not cause cell lethality, as was shown by the ability of these deletants to lose the rescue *LEU2* YEplac181-PUB1 plasmid: streaking cells of corresponding transformants on YPD plates gave rise to 41%, 54% and 38% Leu^-^ clones, respectively (approximately 200 clones were tested for each transformant). This indicates that the observed synthetic lethality was not the consequence of an NMD defect or the increase of nonsense codon readthrough caused by the *UPF1* deletion.

The type of [*PSI*^+^] was important for the synthetic lethality. Simultaneous deletion of *PUB1* and *UPF1* in the same yeast strain carrying weak [*PSI*^+^] variants, [*PSI*^+^]_WS2_ and [*PSI*^+^]_W2_ (Table 2), was not lethal, and corresponding transformants could easily lose the *PUB1 LEU2* rescue plasmid. Among approximately 200 clones growing in nonselective YPD medium, 22% and 36% were Leu^-^, respectively. Thus, the ability to cause synthetic lethality correlated with the strength of the [*PSI*^+^] suppressor phenotype.

### 2.2. The Lack of Pub1 But Not of Upf1 Increases Prion Polymerization of Sup35

The ability of overproduced Sup45 to suppress synthetic lethality of *PUB1* and *UPF1* deletions in the [*PSI*^+^]_S7_ background suggested that this lethality resulted from a depletion of soluble Sup45 caused by its sequestration into Sup35 prion polymers. The deficiency of Sup45 could be aggravated by inhibition of *SUP45* expression by *UPF1* or *PUB1* deletion. However, the levels of Sup45 and Sup35 in the [*psi*^-^] strain were not affected by either *pub1-∆* [23], or *upf1-∆* (Figure S1). Since *pub1-∆* significantly increases the level of Sup35 polymers, it was reasonable to suggest that the deletion of *UPF1* also causes an increase of Sup35 polymerization, and together these deletions increase Sup35 polymerization to the level which is incompatible with cell viability. However, comparison of the amount of Sup35 polymers in the 74-D694 [*PSI*^+^]_S7_ strain carrying either wild-type or deleted *UPF1* did not reveal any effects of this gene on Sup35 polymerization. Importantly, deletions of *UPF2* or *UPF3* genes also did not influence the level of Sup35 prion polymers in this strain (Figure S2).

**Figure 2:**
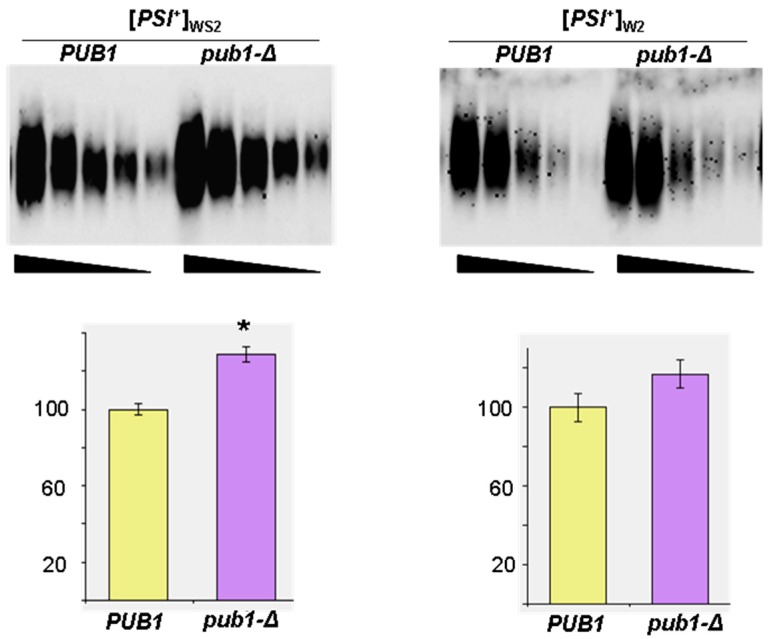
Deletion of *PUB1* slightly increases the levels of Sup35 polymers in cells with weak [*PSI*^+^]. SDD-AGE analysis of polymerized Sup35: The strains were grown in liquid YPD medium; blots were probed with the polyclonal antibody against Sup35NM; equal amounts of total protein from compared cell lysates were serially diluted in twofold decrements; undiluted samples contained ~180 µg of total protein per lane. Four clones of [*PSI*^+^]_WS2_ and [*PSI*^+^]_W2_ derivatives of the 74-D694 strain with deleted (*pub1-Δ*) or without *PUB1* deletion (*PUB1*) grown in liquid YPD were studied, and the abundance of Sup35 polymers in pub1-Δ and PUB1 strains was calculated as described in the legend to Figure 1 and shown as the percent of the level in the strain bearing wild-type *PUB1*± SEM in the histograms. A statistically significant increase in the amount of Sup35 polymers caused by *pub1-Δ* (determined by Student’s *t*-test) and indicated by an asterisk (*) was observed for the strain with [*PSI*^+^]_WS2_ (*p* < 0.05), but not with [*PSI*^+^]_W2_ (*p* > 0.3). Typical blot images are presented.

The role of Pub1 in Sup35 prion polymerization was shown only for one [*PSI*^+^]_S7_ variant [23]. To elucidate whether the effect of *pub1-∆* on Sup35 polymerization was [*PSI*^+^] variant-specific or not, we tested it in the same strain which carried [*PSI*^+^] variants with a weak suppressor phenotype. Analysis of Sup35 polymerization in the strains with [*PSI*^+^]_W2_ and [*PSI*^+^]_WS2_ bearing deletion of the chromosomal *PUB1* demonstrated that *pub1-∆* caused an increase in the amount of Sup35 polymers for [*PSI*^+^]_WS2_, albeit to a lesser extent than for the strain with [*PSI*^+^]_S7_, but had no statistically significant effect on the level of Sup35 polymers in the strain with [*PSI*^+^]_W2_ (Figure 2). 

One can suggest that if the lack of Pub1 stimulates Sup35 prion polymerization, then overproduction of this protein should inhibit it. However, quantitative examination of Sup35 prion polymers in the 74-D694 [*PSI*^+^]_S7_ strain overproducing Pub1 demonstrated that excess of this protein did not decrease the level of Sup35 polymers (Figure S3). Notably that the effect of *pub1-∆* is specific for [*PSI*^+^], since this deletion did not affect polymerization of the Rnq1 protein, which is the protein determinant of the [*PIN*^+^] prion [29,30] (Figure S4).

### 2.3. The Lack of Upf1, But Not of Pub1, Upf2 or Upf3 Increases Sequestration of Sup45 into Sup35 Prion Polymers

**Figure 3:**
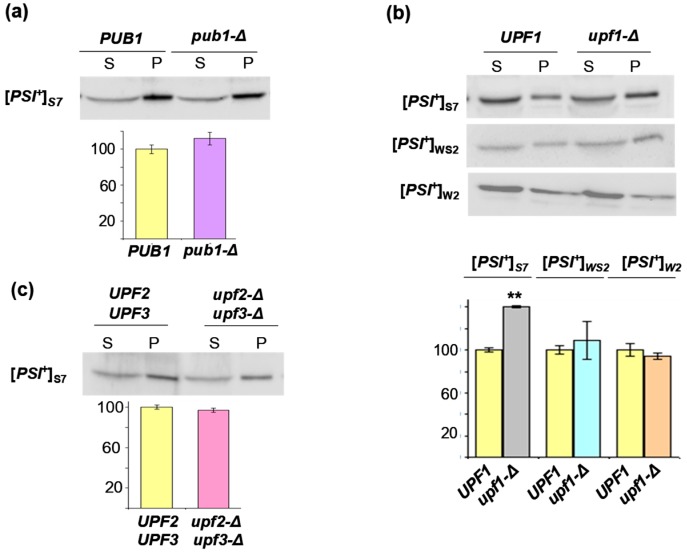
Deletion of *UPF1* but not of *PUB1*, *UPF2* or *UPF3* increases Sup45 aggregation in cells with [*PSI*^+^]_S7_. Cell lysates were fractionated by ultracentrifugation as described in Materials and Methods. Samples were loaded onto the gel in a volume corresponding to equal amounts of starting lysates, which contained ~200 µg of soluble protein. Blots were probed with polyclonal antibody against Sup45 and levels of Sup45 in fractions were determined by densitometric analysis of blots. Four clones of each strain were analyzed. S, soluble fraction; P, pellet. The relative abundances of Sup45 in these fractions estimated by densitometry of blots, were calculated as ratios of its signal intensity in the pellet fraction versus the sum of signal intensities in the pellet and soluble fractions and is shown on the histograms as the percent of the level in the strain wild-type for analyzed gene. Typical blot images are presented. The statistical significance of differences in the amount of aggregated Sup45 in compared strains was estimated by Student’s *t*-test. (**a**) Deletion of *PUB1* (*pub1-Δ*) does not cause a statistically significant increase in the amount of aggregated Sup45 (*p* > 0.08), (**b**) deletion of *UPF1* (*upf1-Δ*) causes a statistically significant (*p* < 0.001) increase of the amount of aggregated Sup45 in cells with [*PSI*^+^]_S7_, indicated by two asterisks (**), but not in cells with either [*PSI*^+^]_WS2_ (*p* > 0.2) or [*PSI*^+^]_W2_ (*p* > 0.7), (**c**) simultaneous deletion of *UPF2* (upf2-Δ) and *UPF3* (upf3-Δ) in [*PSI*^+^]_S7_ cells does not influence Sup45 aggregation (*p* > 0.6).

It was demonstrated earlier that in [*PSI*^+^] cells Sup45 is found mostly in the aggregated state, possibly due to its recruitment by Sup35 prion polymers [24,25], though other studies have not confirmed [*PSI*^+^]-dependent co-aggregation of Sup35 and Sup45 [6,31]. If the Sup35 prion polymers sequester Sup45, then the elevation of their level should further increase aggregation of Sup45. However, sedimentation analysis of lysates of the [*PSI*^+^]_S7_ cells with wild-type and deleted *PUB1* did not show a statistically significant difference in the amount of aggregated Sup45 (Figure 3A). Therefore, the 2-fold increase in the level of Sup35 polymers caused by *pub1-∆* in the cells grown in YPD [23] was not sufficient to secure a noticeable difference of co-aggregated Sup45 in the strains with wild-type and deleted *PUB1* grown in the same medium. 

## 3. Discussion

In this work we demonstrate the phenomenon of a triple synthetic lethal interaction in yeast; namely, that the combination of the *PUB1* and *UPF1* deletions with the [*PSI*^+^] prion is lethal, albeit it was observed only for the strain bearing the [*PSI*^+^]_S7_ variant, manifesting a strong suppression phenotype. We also show that the reason for this lethality is inactivation of Sup45 due to depletion of its soluble and functionally active form caused by sequestration of this protein into Sup35 prion polymers. However, in contrast to *upf1-∆*, the deletion of *PUB1* did not cause a noticeable increase of Sup45 aggregation, though, depending on growth conditions, its absence caused up to a 2-fold increase in the amount of Sup35 polymers [23]. Unlike *PUB1*, the *UPF1* gene did not affect Sup35 prion polymerization; however, it was involved in maintaining a normal level of soluble Sup45 in cells with strong [*PSI*^+^], since its deletion in these cells resulted in an approximately 1.5-fold increase of aggregated Sup45. Importantly, though the increase in the level of Sup35 polymers caused by *pub1-∆* on its own did not cause observable changes in the aggregation of Sup45, it did increase the aggregation of this protein when *pub1-∆* was combined with *upf1-∆*. Indeed, in this strain a 2-fold increase in the amount of Sup35 polymers and a 1.5-fold increase in Sup45 co-aggregation resulted in a 3-fold increase in the aggregation of Sup45 as compared to the strain with wild-type *PUB1* and *UPF1*. Remarkably, it was shown earlier that [*PSI*^+^] can inactivate the Sup45 translation termination factor, since deleting one copy of the *SUP45* gene in a [*PSI*^+^] but not in a [*psi*^−^] diploid strain caused a noticeable inhibition of cell growth and blocked sporulation [15]. Thus, since a 2-fold decrease of Sup45 amounts is harmful to [*PSI*^+^] cells, it is not surprising that a further 3-fold depletion of soluble Sup45 can be lethal.

Two mechanisms can explain the ability of Pub1 to restrict Sup35 polymerization: (i) Pub1 binds to the ends of Sup35 polymers to restrain their further elongation; and (ii) Pub1 forms complexes with monomeric Sup35, thus inhibiting its ability to join to the ends of a growing polymer. The latter possibility is supported by the observation that Pub1∆C, lacking the short Q-rich C-terminal region, which is critical for its co-polymerization with Sup35 and contains the major site for interaction with monomeric Sup35, suppresses Sup35 polymerization, though less efficiently than full-length Pub1. Most probably, this ability can be attributed to the Pub1 internal low-affinity site for interaction with monomeric Sup35 [23]. Besides, the observation that the lack of the Pub1 protein, which does not interact with monomeric Rnq1 but copolymerizes with it in [*PIN*^+^] cells [32], does not influence Rnq1 polymerization, is also in line with this suggestion. Importantly, this mechanism explains [*PSI*^+^] variant-dependent effects of *pub1-∆* on Sup35 polymerization efficiency. It is known that cells with strong [*PSI*^+^] contain much less soluble Sup35 than cells with weak variants of this determinant [9,33], and therefore, Pub1 can bind a greater proportion of soluble Sup35 in cells with strong [*PSI*^+^] than in cells with weak [*PSI*^+^]. If this is correct, the lack of Pub1 should ensure the most profound effect on Sup35 polymerization in cells with strong [*PSI*^+^] variants. Furthermore, according to the same considerations, excess Pub1 does not decrease the levels of Sup35 polymers in strong [*PSI*^+^] due to insufficient amount of Sup35 monomers available for interaction with Pub1.

Interestingly, it was shown recently that most [*PSI*^+^] variants, which appeared in the absence of Upf proteins, can be eliminated by restoration of the normal levels of these proteins. To explain this effect, it was proposed that inhibition of [*PSI*^+^] prion propagation by Upf proteins may be due to their interaction with soluble Sup35, which distracts this protein from polymerization or, alternatively, with polymerized Sup35, which blocks adding Sup35 monomers to the ends of growing polymers [34]. However, here we show that in contrast to *pub1-∆*, deletion of any of the *UPF* genes does not increase the amount or the size of Sup35 polymers, indicating that at least in cells with [*PSI*^+^] generated in the presence of wild-type *UPF* genes, Upf proteins are not involved in the process of Sup35 polymerization.

**Figure 4:**
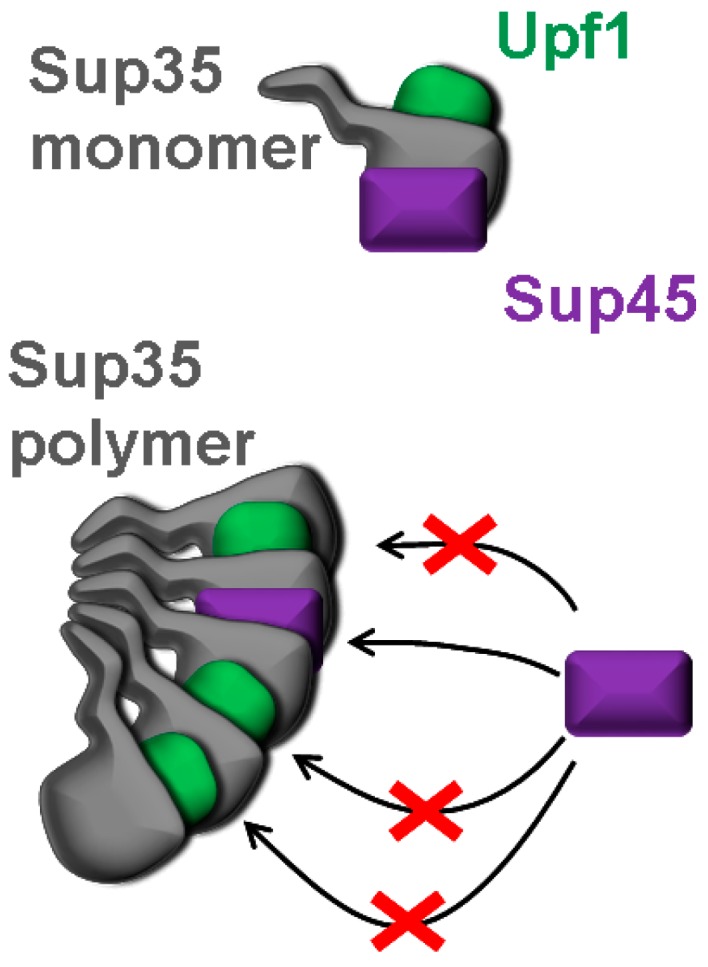
Schematic representation of the suggested mechanism mediating the effect of Upf1 on binding of Sup45 to polymeric Sup35. Upf1 and Sup45 interact with monomeric Sup35 independently. Sup45 binds to Sup35 involved in a polymer only if Sup35 is not bound to Upf1.

Though, unlike Pub1, Upf1 did not influence Sup35 polymerization, it controlled the level of soluble Sup45 by inhibiting binding of Sup45 to Sup35 polymers, which could be due to its interaction with these polymers. Importantly, the ability of Upf1 to interact with Sup35 polymers was supported by observation of [*PSI*^+^]-dependent co-sedimentation of these proteins [24], as well as co-localization of their fusions with alternative fluorescent proteins [34]. Nevertheless, the effect of Upf1 on interaction of Sup45 with Sup35 polymers seems surprising, since it is known that only Upf2 and Upf3, but not Upf1 compete with Sup45 for binding to monomeric Sup35, which agrees with a spatial separation of corresponding binding sites in Sup35. Indeed, it was shown that Upf1 interacts with Sup35 through a proximal site in its C-terminal domain, while Upf2, Upf3 and Sup45 bind to the overlapping sites located in a distal region of this Sup35 domain [27]. Thus, it remains to suggest that Upf proteins interact differently with monomeric and polymeric Sup35. It is probable that the site for Upf1 binding in Sup35 involved in a polymer is exposed, while the site for Upf2, Upf3 and Sup45 is not, and the lack of Upf1 makes this Upf2/Upf3/Sup45-specific site available for interaction with Sup45 (Figure 4). This also explains the inability of *UPF2* and *UPF3* deletions to influence binding of Sup45 to polymerized Sup35.

Proteins whose absence affects [*PSI*^+^] formation, propagation and/or phenotypic manifestation can be divided into two classes. The first class involves cytosolic chaperones of the Hsp40, Hsp70 and Hsp100 families as well as the chaperone sorting factor Cur1 [19,35,36]. The second class includes functionally unrelated non-chaperone proteins, such as vacuolar proteases PrA and PrB [22], Upf1/2/3 proteins controlling NMD and nonsense codon readthrough [34], as well as Siw14 and Arg82, enzymes involved in the inositol polyphosphate biosynthetic pathway [21]. Mechanisms of action of these proteins remain elusive, with the exception of PrA and PrB proteases, which cleave off an important part of the Sup35 prion-forming domain.

Notably, besides the anti-prion systems counteracting [*PSI*^+^] formation, yeast cells contain systems preventing [*PSI*^+^] cytotoxicity, which are also based on both chaperone and non-chaperone proteins. One of these systems is based on the nascent polypeptide-associated complex representing a highly conserved triad of proteins that bind near the ribosome exit tunnel. It was shown that deletion of subunits of this complex rescues toxicity associated with the strong [*PSI*^+^] prion, which can be explained by changes in chaperone balance and distribution, whereby the folding of the prion protein is improved and the prion is rendered nontoxic [37]. Another chaperone-assisted [*PSI*^+^] detoxification system is based on the Hsp40 Sis1 chaperone [38]. The mechanism of the toxicity, which is rescued by Sis1, is not yet clear, but most probably it is not related to Sup45 depletion. Other [*PSI*^+^] anti-toxic systems described involve non-chaperone proteins. One of them, revealed here, consists of two proteins, Pub1 and Upf1; the former saves the cell from excessive Sup35 polymerization, while the latter alleviates binding of Sup45 to Sup35 polymers. One more such system involves the actin assembly protein Sla2, whose protective effect is unlikely to involve sequestration of Sup45 into prion aggregates [13]. Thus, it is possible that at least two proteins, Sis1 and Sla2, alleviate [*PSI*^+^] toxicity by preventing sequestration of essential cellular components other than Sup45 into prion aggregates. This suggests that different [*PSI*^+^] detoxification systems may protect the cell from the defects of various essential processes not related to translation. Indeed, Sup35 was shown to possess essential functions unrelated to its role in the translation termination [39,40], which can be compromised by its prion aggregation.

Finally, it should be stressed that although the role of [*PSI*^+^] in yeast biology is still unclear, it is possible that even if most commonly appearing [*PSI*^+^] variants are harmful, some of them can be beneficial and due to this, yeast has developed special systems for self-protection from the deleterious side effects of this prion.

## 4. Materials and Methods

### 4.1. Yeast Strains and Growth Conditions

All experiments described in this study were performed with the use of the [*psi*^−^][*pin*^−^] derivative of the *S. cerevisiae* strain 74-D694 (*MAT*a *ura3-52 leu2-3,112 trp1-289 his3-∆200 ade1-14*), as well as its variants carrying [*PIN*^+^] and either strong [*PSI*^+^], originally present in this strain and designated here as [*PSI*^+^]_S7_ [41] or weak [*PSI*^+^]_WS2_ and [*PSI*^+^]_W2_ which were generated in the [*psi*^−^][*PIN*^+^] background by transient overproduction of Sup35 and selected by the ability to suppress the *ade1-14*^UGA^ mutation [42]. The construction of genetically-modified variants of this strain is described in the next section. Yeast were grown at 30 °C in rich (YPD, 1% yeast extract, 2% peptone, 2% glucose) or synthetic (SC, 0.67% yeast nitrogen base, 2% glucose supplemented with appropriate amounts of the required amino acids or bases) media. All growth assays were made in triplicate.

### 4.2. Plasmids and Nucleic Acid Manipulation

Plasmids used in this study are presented in Table 3. To generate the plasmids pRS316-PUB1 and YEplac181-PUB1, the *PUB1* gene harboring the EcoRI-XbaI fragment of YEplac195-PUB1 was inserted into the same sites of the pRS316 and YEplac181 plasmids, respectively. The EcoRI-XbaI fragment of YEplac195-PUB1ΔC was inserted into the same site of pRS316 to generate the pRS316-PUB1ΔC plasmid. To construct the pRS315-UPF1 and YEplac181-UPF1 plasmids, the *UPF1* gene harboring the PstI-PvuII fragment of YEplac112-UPF1 was inserted into the PstI and SmaI sites of the pRS315 and YEplac181 plasmids, respectively. The *UPF1* gene was disrupted in the 74-D694 [*PIN*^+^][*PSI*^+^]_S7_ strain using the *upf1*::*URA3* disruption cassette, as described in Reference [43]. The *upf2::URA3* gene disruption cassette was obtained by PCR amplification using the primers 5′-GTGTACTGGAACGGTCCAATA-3′ and 5′-ATACACTGGCAGTTTGCTCCA-3′ and the genomic DNA of the Y41 strain (*MAT*a *his4-38 SUF1-1 ura3-52 leu2-3 trp1-1 UPF2::URA3*), which is the *UPF2* disruption derivative of the PLY18 strain [43]. This cassette was used to disrupt *UPF2* in the 74-D694 [*PIN*^+^][*PSI*^+^]_S7_ strain. Similarly, the *upf3::kanMX* gene disruption cassette, obtained by PCR amplification using primers 5′-CCCCATGTAAATCATCCAAT-3′ and 5′-TGGAGTCATCTTTCTTCATG-3′, and the genomic DNA of the *upf3-Δ* derivative of the BY4742 strain (*MAT*α *his3-Δ1 leu2-Δ0 lys2-Δ0 ura3-Δ0 upf3::kanMX) obtained from* EUROSCARF, was used to select the G418-resistant *UPF3* disruptant of the 74-D694 [*PIN*^+^][*PSI*^+^]_S7_ strain. The *PUB1* disruptant of the 74-D694 [*PIN*^+^][*PSI*^+^]_S7_ strain and the procedures for the *PUB1* gene disruption in 74-D694 derivatives with [*PSI*^+^]_WS2_ and [*PSI*^+^]_W2_ using the *pub1::TRP1* disruption cassette were the same as described earlier [23]. Disruption of the above-mentioned genes was verified by PCR analysis. 

### 4.3. Electrophoresis and Blotting

SDS-PAGE was performed according to the standard protocol in 10% polyacrylamide gels and SDD-AGE (Semi denaturing detergent–agarose gel electrophoresis) as described previously [7,44]. Protein loads were equalized for each gel. For the SDD-AGE analysis of amyloid polymers we used horizontal 1.8% agarose gels in the Tris-Acetate-EDTA (TAE) buffer with 0.1% SDS. Lysates were incubated in sample buffer (0.5 × TAE, 2% SDS, 5% glycerol and 0.05% Bromophenol Blue) for 5 min at room temperature. After electrophoresis, proteins were transferred from gels to nitrocellulose membrane sheets (Thermo Scientific, Waltham, MA, USA) by vacuum-assisted capillary blotting for 8 h (agarose gels), or electrophoretically (polyacrylamide gels). Bound antibody was detected using the enhanced chemiluminescence (ECL) West Dura system (Thermo Scientific, Waltham MA, USA). It should be noted that detergents (SDS or sarcosyl) in non-boiled samples increase degradation of Sup35 monomers. This can result in the absence of Sup35 monomer bands in SDD-AGE gels. Rabbit polyclonal antibodies against yeast Sup35NM (Sup35 lacking the C-terminal domain responsible for translation termination activity), Sup45 [39,45] and Pub1 [32] were used. Densitometry measurements were performed using ImageJ software.

### 4.4. Preparation and Fractionation of Yeast Cell Lysates

Yeast cells grown in liquid selective media to OD_600_ of 2.5 were harvested, washed in water and disrupted by beating with glass beads (Bullet Blender, Next Advance, Troy, NY, USA) in buffer A: 30 mM Tris-HCl, pH 7.4, 150 mM NaCl, 1 mM dithiothreitol with 10 mM phenylmethylsulfonyl fluoride and CompleteTM protease inhibitor cocktail (Roche Applied Science, Indianapolis, IN, USA) to prevent proteolytic degradation. After centrifugation of crude lysates at 1500× *g* for 4 min, cell debris containing glass beads was washed in buffer A, containing 1% Triton X-100 or 1% SDS, if polymers of Sup35 and Rnq1 were analyzed by SDD-AGE. To analyze the content of soluble and aggregated Sup45 by centrifugation, cells were grown to OD_600_ of 2.0. The lysates were prepared in buffer A, crowding agent, Ficoll PM400 at a concentration close to the physiological concentration of macromolecules (200 mg/mL) and 20 mM EDTA dissociating ribosomes to subunits. Lysates (0.05 mL) were underlaid with the same volume of 30% sucrose pads made in buffer A and centrifuged at 100,000× *g*, 4 °C for 90 min. Pellets were resuspended in volumes equal to the volumes of the ultracentrifuged lysates. The resulting supernatant and pellet fractions were analyzed by Western blotting using antibodies against Sup45.

### 4.5. Determination of the Efficiency of Nonsense Codon Readthrough

To measure the efficiency of nonsense codon readthrough, plasmids of a pDB series carrying tandem *Renilla* and firefly luciferase genes separated by a single in-frame UGA(C) codon or a corresponding sense codon control were used [28,52]. Assays were performed with a dual luciferase reporter assay system (Promega, Madison, WI, USA), as described [53] with minimal modifications using a Glomax 20/20 luminometer (Promega, Madison, WI, USA). All assays were repeated three times. The readthrough in each strain is expressed as the ratio of firefly luciferase activity/*Renilla* luciferase activity (nonsense codon between luciferase genes) divided by the ratio of firefly luciferase activity/*Renilla* luciferase activity (sense codon between luciferase genes). For further details, see Reference [54].

## Figures and Tables

**Table 1:** Overproduction of Upf1, Pub1, Pub1∆C, Sup45 and Sup35C rescues the [*PSI*^+^]_S7_ *pub1-∆ upf1-∆* cells from lethality.

Plasmid	Rescue Plasmid Loss (%)	Suppression of Synthetic Lethality
Multi-*UPF1*	36	+
CEN-*UPF1*	52	+
*Multi-*PUB1*	86	+
*CEN-*PUB1*	87	+
*Multi-*pub1-∆C*	36	+
*CEN- *pub1-∆C*	50	+
Multi-*SUP45*	84	+
CEN-*SUP45*	53	+
Multi-*sup35-C*	38	+
CEN- *sup35-C*	0	−
Empty vector	0	−

Multi and CEN, multicopy and centromeric plasmids, respectively. Transformants carried two plasmids, the rescue plasmid with wild-type *PUB1* and with either *LEU2* or *URA3* as selectable markers (YEplac181-PUB1 or YEplac195-PUB1, respectively) and the other with the tested gene and appropriate selectable marker. As a control, the empty vectors YEplac195 or YEplac181 with either *URA3* or *LEU2*, respectively, were used. Transformants were streaked on SC medium selective for the marker of plasmid carrying the tested gene. For each transformant, more than 100 clones growing up were examined. The percentage of clones that lost the rescue plasmid, was calculated. + and − indicate ability or inability of the plasmid to suppress synthetic lethality, respectively, which is concluded from the ability to lose the rescue plasmid. * Transformant, the growth rate of which was studied (Figure 1b).

**Table 2:** Efficiency of nonsense codon readthrough caused by different [*PSI*^+^] variants.

[*PSI*^+^] Variant	% Readthrough
[*PSI*^+^]_S7_	6.1 ± 0.4
[*PSI*^+^]_WS2_	2.1 ± 0.1
[*PSI*^+^]_W2_	1.5 ± 0.2

The UGAC stop signal was used for measurement, which shows the highest readthrough among all stop codons [23,28]. Percent readthrough is expressed as the mean ± SEM.

**Table 3:** Plasmids.

Plasmids	Characteristics	Source
YEplac181	Multicopy *LEU2* plasmid	[46]
YEplac181-PUB1	Multicopy *LEU2* plasmid harboring the *PUB1* gene	This work
YEplac181-SUP35C	Multicopy *LEU2* plasmid encoding Sup35C	[47]
Yeplac181-UPF1	Multicopy *LEU2* plasmid harboring the *UPF1* gene	This work
YEplac195	Multicopy *URA3* plasmid	[46]
YEplac195-PUB1	Multicopy *URA3* plasmid harboring the *PUB1* gene	[32]
YEplac195-PUB1ΔC	Multicopy *URA3* plasmid encoding Pub1ΔC	[23]
YEplac195-SUP45	Multicopy *URA3* plasmid harboring the *SUP45* gene	[48]
YEplac112-UPF1	Multicopy *TRP1* plasmid harboring the *UPF1* gene	[49]
pRS315	Centromeric *LEU2* plasmid	[50]
pRS315-SUP35C	Centromeric *LEU2* plasmid encoding Sup35C	[40]
pRS315-SUP45	Centromeric *LEU2* plasmid harboring the *SUP45* gene	[45]
pRS315-UPF1	Centromeric *LEU2* plasmid harboring the *UPF1* gene	This work
pRS316	Centromeric *URA3* plasmid	[50]
pRS316- PUB1	Centromeric *URA3* plasmid harboring the *PUB1* gene	This work
pRS316- PUB1ΔC	Centromeric *URA3* plasmid encoding Pub1ΔC	This work
pEMBLyex4(ΔLEU2_d_)-3ATG	Multicopy *URA3* plasmid encoding Sup35C	[51]
pPUB1::TRP1	Plasmid encoding *pub1::TRP1* disruption cassette	[23]
pKOM	Plasmid encoding *upf1::URA3* disruption cassette	[43]

## Supplementary Materials

Supplementary Materials can be found at http://www.mdpi.com/1422-0067/19/11/3663/s1.
