# Supplementary material for: The Pub1 and Upf1 Proteins Act in Concert to Protect Yeast from Toxicity of the [PSI+] Prion
Source: Int J Mol Sci. 2018 Nov 20;19(11):3663. doi: 10.3390/ijms19113663 (PMC6275000; doi:10.3390/ijms19113663)
Supplement: Supplementary file 1 [file ijms-19-03663-s001.pdf]

Supplementary Materials: The Pub1 and Upf1 proteins act in concert to protect yeast from toxicity of the [*PSI*$^+$] prion

Valery N. Urakov, Olga V. Mitkevich, Alexander A. Dergalev, Michael D. Ter-Avanesyan

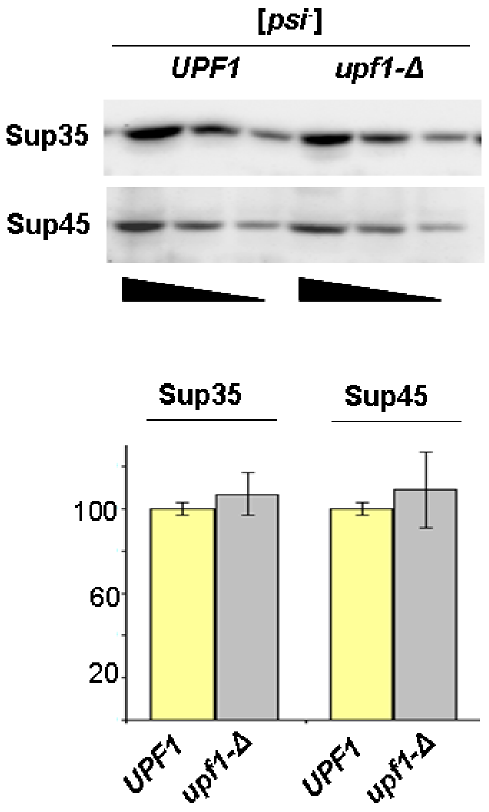

Figure S1. Deletion of *UPF1* does not influence the levels of Sup35 and Sup45 in the [*psi*$^-$] cells. SDS-PAGE analysis of the amount of Sup35 and Sup45. Lysates were prepared from the cells harvested at the late exponential phase of growth in YPD. Equal amounts of total protein from the cell lysates of the 74-D694 [*psi*$^-$] strain (*UPF1*) and its derivative with deleted *UPF1* (*upf1*-Δ) were serially diluted with two-fold increments. Four clones of the *UPF1* and *upf1*-Δ strains were analyzed. Blots were probed with the polyclonal antibodies against Sup35NM a/nd Sup45. The densitometry of corresponding blots demonstrated that the difference of Sup35 and Sup45 abundances between the *UPF1* and *upf1*-Δ strains estimated by the Student's t-test is statistically insignificant ($P > 0.6$ and $P > 0.4$, respectively). The amount of Sup35 or Sup 45 is presented as the percentage of their amount in the *UPF1* strain. Typical blot images are presented.

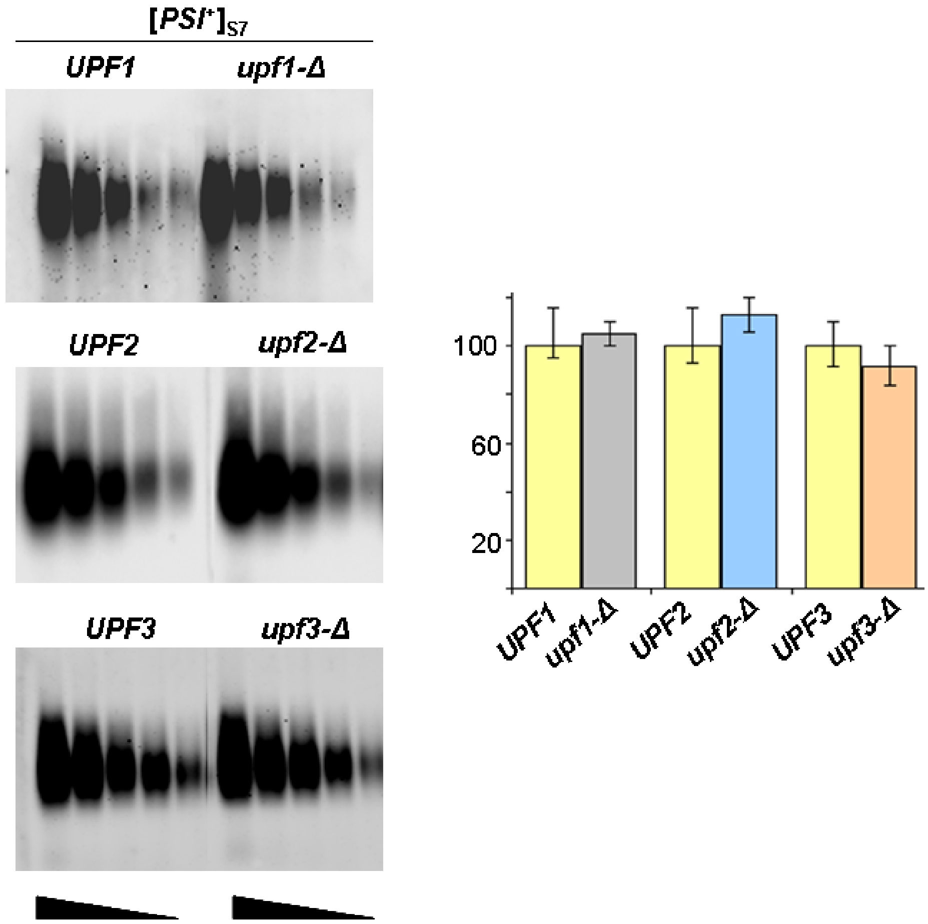

Figure S2. Deletions of the *UPF1/2/3* genes do not influence the [*PSI*$^+$]$_{S7}$-dependent Sup35 prion polymerization. The effect of *upf1*-Δ, *upf2*-Δ and *upf3*-Δ on polymerization of Sup35 in cells of the 74-D694 strain with strong [*PSI*$^+$]$_{S7}$ grown in liquid YPD. The SDD-AGE analysis of polymerized Sup35. Four clones of each strain were analyzed. Blots were probed with the polyclonal antibody against Sup35NM. Equal amounts of total protein from compared cell lysates were serially diluted with twofold increments. Determination of the amount of Sup35 polymers in the *upf1/2/3*-Δ deletants with [*PSI*$^+$]$_{S7}$ and in the isogenic control strain with wild type *UPF* genes, calculated after densitometry of blots and estimated by the Student's t-test, demonstrates the absence of statistically significant differences ($P > 0.5$ for all compared pairs of strains). The amount of Sup35 polymers is presented as the percent of the level in the strain bearing wild-type of analyzed gene. Typical blot images are presented.

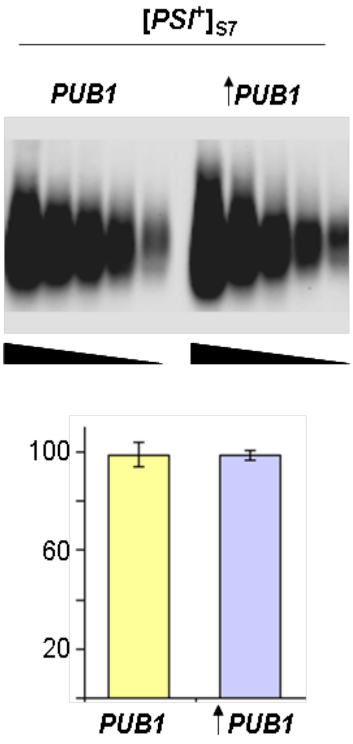

Figure S3. Overexpression of *PUB1* does not affect polymerization of Sup35 in cells with [*PSI*+]$_{S7}$. Four transformants of both 74-D694 [*PSI*+]$_{S7}$ with the wild type chromosomal *PUB1* carrying the *PUB1* multicopy plasmid YEplac195-PUB1 (↑*PUB1*) and the same strain carrying the empty vector YEplac195 (*PUB1*) were studied and the relative abundance of Sup35 polymers in the *PUB1* and ↑*PUB1* transformants, determined as described in legend to Figure 1 demonstrated the absence of statistically significant difference ($P > 0.7$). The amount of Sup35 polymers is presented as the percent of the level in the *PUB1* strain. Typical blot images are presented.

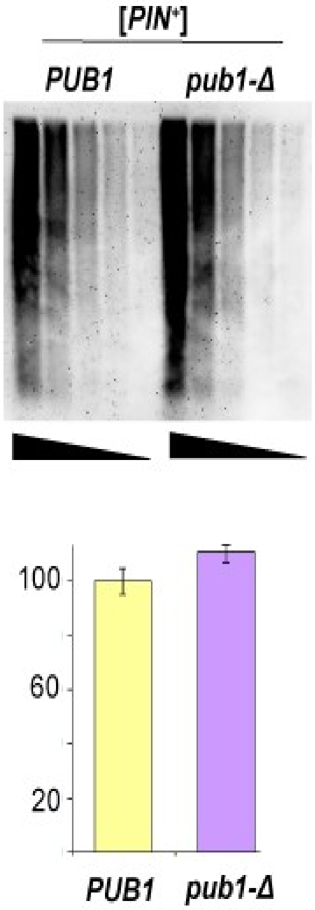

Figure S4. Deletion of *PUB1* does not affect the polymerization of Rnq1 in [*PIN*$^+$] cells. SDD-AGE analysis of polymerized Rnq1 in cells of the 74-D694 [*PIN*$^+$][*PSI*$^+$]$_{S7}$ strain with wild type (*PUB1*) and deleted *PUB1* (*pub1*-Δ) grown in YPD. Three clones of each strain were analyzed. Blots were probed with the polyclonal antibody against Rnq1. Equal amounts of total protein from compared cell lysates were serially diluted with twofold increments. Determination of the amount of the Rnq1 polymers in strains with deleted and wild type *PUB1*, calculated as described in legend to Figure 1, demonstrated the absence of statistically significant difference ($P > 0.3$). Typical blot images are presented.
